# The long winding road to the safer glucocorticoid receptor (GR) targeting therapies

**DOI:** 10.18632/oncotarget.28191

**Published:** 2022-02-18

**Authors:** Ekaterina A. Lesovaya, Daria Chudakova, Gleb Baida, Ekaterina M. Zhidkova, Kirill I. Kirsanov, Marianna G. Yakubovskaya, Irina V. Budunova

**Affiliations:** ^1^Deparment of Chemical Carcinogenesis, Institute of Carcinogenesis, N.N. Blokhin NMRCO, Moscow, Russia; ^2^Department of Oncology, I.P. Pavlov Ryazan State Medical University, Ryazan, Russia; ^3^Department of Dermatology, Northwestern University, Chicago, IL, USA; ^4^Deparment of General Medical Practice, RUDN University, Moscow, Russia

**Keywords:** glucocorticoid receptor, glucocorticoids, SEGRAM, REDD1, drug repurposing

## Abstract

Glucocorticoids (Gcs) are widely used to treat inflammatory diseases and hematological malignancies, and despite the introduction of novel anti-inflammatory and anti-cancer biologics, the use of inexpensive and effective Gcs is expected to grow. Unfortunately, chronic treatment with Gcs results in multiple atrophic and metabolic side effects. Thus, the search for safer glucocorticoid receptor (GR)-targeted therapies that preserve therapeutic potential of Gcs but result in fewer adverse effects remains highly relevant. Development of selective GR agonists/modulators (SEGRAM) with reduced side effects, based on the concept of dissociation of GR transactivation and transrepression functions, resulted in limited success, and currently focus has shifted towards partial GR agonists. Additional approach is the identification and inhibition of genes associated with Gcs specific side effects. Others and we recently identified GR target genes REDD1 and FKBP51 as key mediators of Gcs-induced atrophy, and selected and validated candidate molecules for REDD1 blockage including PI3K/Akt/mTOR inhibitors. In this review, we summarized classic and contemporary approaches to safer GR-mediated therapies including unique concept of Gcs combination with REDD1 inhibitors. We discussed protective effects of REDD1 inhibitors against Gcs–induced atrophy in skin and bone and underlined the translational potential of this combination for further development of safer and effective Gcs-based therapies.

## INTRODUCTION

Glucocorticoids (Gcs) are among the most frequently used anti-inflammatory and anti-lymphoma drugs. Synthetic Gcs were first introduced into clinical practice in 50-s, and since then, more than 30 Gcs were approved for systemic and topical use. Despite the recent successful development of novel immunosuppressive and anti-cancer drugs and novel biologics targeting specific disease-related signaling pathways, very effective and inexpensive Gcs remain widely used. Overall, the global Gcs market was ~ $4.5 billion in 2020 with the topical Gcs as a dominant segment, and it is expected that market will continue to steadily grow. The biological effects of Gcs are mediated by the glucocorticoid receptor (GR), a transcription factor that regulates the expression of thousands of target genes, and plays an important role in the regulation of glucose, lipid and protein metabolism, stress response, cell proliferation and apoptosis, and inflammation. Unfortunately, chronic treatment with Gcs results in multiple metabolic, atrophic and other adverse effects that became apparent starting from their early use. The increased understanding of GR molecular biology and the mechanisms underlying therapeutic and side effects of Gcs, powered a tremendous effort of pharmaceutical companies and academia directed toward the development of safer GR-targeted therapies. In this review, we discuss modern understanding of molecular peculiarities of GR function providing different approaches to these safer therapies including the search for alternative (frequently non-steroidal) GR ligands with preserved therapeutic activities but reduced side effects; and the search for “tissue protectors” (based on the drug repurposing of FDA-approved and experimental drugs) to use in combination with Gcs.

### Effects of glucocorticoids are mediated by the glucocorticoid receptor

Gcs are steroid hormones mostly generated in the adrenal cortex, even though some are synthesized locally from precursors from cholesterol metabolites such as pregnenole by different enzymes including CYP11A1 [1, 2]. The major natural glucocorticoid in humans is cortisol; it is converted from inert precursor cortisone by 11β-hydroxysteroid dehydrogenase type 1 (11βHSD1). The reverse conversion from biologically active cortisol to non-active cortisone is mediated by 11βHSD2.

Key functions of Gcs include regulation of glucose, protein and lipid metabolism, cell proliferation and differentiation, development, stress response, apoptosis, immune response and inflammation [3, 4].

Gcs exert their effects via the glucocorticoid receptor (GR, NR3C1), a well-known transcription factor (TF) from the superfamily of nuclear hormone receptors [3, 5]. In inactive conformation it resides in the cytoplasm bound to the molecular chaperones—heat shock proteins and immunophilins [4]. Upon stimulation by Gcs, GR undergoes phosphorylation, homodimerization, and translocates to the nucleus. Activation of gene expression (transactivation, TA) requires GR homodimer binding to the palindromic Gc responsive elements (GRE) in gene promoters. Negative gene expression regulation (transrepression, TR) is mediated via diverse mechanisms including binding of GR to less conserved negative GREs or by binding of GR monomer to other TFs including pro-inflammatory NF-kB, AP-1, IRF, (interferon-regulated factors), STATs , thus blocking their activity [6–9].

It has been well accepted in the field that TR is an important mechanism underlying therapeutic anti-inflammatory and anti-lymphoma effects of Gcs [10]. At the same time, mediated by GR homodimer TA regulates GR signaling linked to gluconeogenesis, lipid and protein catabolism, and often mediates the development of atrophic effects in different tissues (such as skin and muscle atrophy) as well as some metabolic adverse effects (hyperglycemia, steroid-induced diabetes) [7, 11–13].

### Glucocorticoids as therapeutic agents

Isolation and crystallization of natural Gcs in 1920–1930s led to the successful synthesis of cortisone in 1947 [14]. In 1948 the first patient with rheumatoid arthritis was treated with cortisone, and in 1952 hydrocortisone was used for the first time to treat atopic dermatitis. Since then, more than 30 Gcs were approved for systemic and topical clinical use, including betamethasone, budesonide, cortisone, dexamethasone (Dex), hydrocortisone, methylprednisolone, prednisolone, prednisone, triamcinolone. Their wide use in clinical practice is based on their strong anti-inflammatory activity and anti-proliferative/pro-apoptotic effects important for anti-cancer activity. Indeed, Gcs inhibit expression of a large set of inflammatory cytokines and chemokines IL-1, IL-2, IL-12, IL-18, TNF-α, INF-γ, GM-CSF, and others, as well as central regulators of cell cycle – Cyclins and CDKs, [6, 14, 15].

Despite a recent successful development of novel anti-inflammatory and anti-cancer therapies targeting specific cytokines and growth factors/pathways, the inexpensive and very efficient Gcs are still widely used for the treatment of millions of patients with allergies, chronic inflammatory and autoimmune diseases such as asthma, rheumatoid arthritis (RA), ulcerative colitis/Crohn disease, multiple sclerosis, inflammatory and hyperproliferative skin diseases including atopic dermatitis and psoriasis, different skin rashes, itches [3, 16, 17]. Gcs are also extensively used for the treatment of different forms of ocular inflammation, macular edema, and macular degeneration and due to their anti-angiogenic properties for prevention of neovascularization in the eye [3, 18–21]. In addition, they are also an important part of the postoperative patient management and immunosuppressive combination therapies in organ transplant recipients.

Gcs have been an effective component of anti-lymphoma therapies due to their anti-proliferative, pro-apoptotic and anti-angiogenic activities [22]. Synthetic Gcs, such as Dex, are routinely included in chemotherapy protocols of acute lymphoblastic leukemia, chronic lymphocytic leukemia, multiple myeloma, Hodgkin’s and non-Hodgkin’s lymphoma [23].

In case of epithelial cancers, Gcs are mostly used as a palliative therapy to reduce the adverse effects of chemotherapy: to increase appetite, decrease nausea (for some chemotherapeutic regimens that include cisplatin, Gcs are first-line antiemetics), weight loss, reduce fatigue, severe skin rashes typical for EGF and folate inhibitors. Gcs are also applied in abatement of pain associated with bone metastasis by inhibiting the secretion of prostaglandins [24]. Even more, Gcs provided modest therapeutic benefit in early stage prostate cancer and demonstrated potential for use in ER-positive breast cancers due to GR inhibition of E2-mediated cell proliferation [25–28].

Currently Gcs market was estimated for more than $4 billion with the predomination of topical Gcs, which represent up to 60–80% of total dermatological and ophthalmological products sold [29]. It is expected that market will continue to grow by ~ 3.5–4% yearly due to the increasing incidence of chronic diseases and growing geriatric population preferentially treated with Gcs for inflammatory diseases [30].

### Major metabolic and atrophic adverse effects of glucocorticoids

Despite well-known Gcs efficacy as anti-inflammatory and anti-lymphoma drugs, chronic treatment is hampered by multiple adverse effects, most of which became apparent starting from early use of Gcs in 60-s. The list of Gc-induced adverse effects includes the whole spectra of complications: from hypertension and cardiovascular complications (including increased coagulation and vein thrombosis), glaucoma, metabolic and atrophic complications to cognitive and mood disorders [3, 22, 31–38].

### Metabolic syndrome: Gc-induced hyperglycemia, insulin resistance, diabetes and fat metabolism

One of the fundamental physiological functions of Gcs is to oppose the effects of insulin and enhance the liver production of glucose [22, 34, 35]. Gcs decrease rate-limiting insulin receptor signaling molecules and reduce insulin-mediated increase in blood flow to muscles, simultaneously promoting gluconeogenesis in the liver via upregulation of enzymes tyrosine aminotransferase (TAT), glucose-6-phosphatase (G6P) and phosphoenolpyruvate carboxykinase (PEPCK) [22, 34, 39]. Activation of these mechanisms lead to a deregulated carbohydrate metabolism, hyperglycemia, and in more severe cases to steroid-induced diabetes. Gcs also control lipid metabolism, preadipocyte maturation, and distribution and accumulation of fat in different fat depots [40–45]. Chronic treatment with Gcs results in fat redistribution, truncal (central) and visceral obesity accompanied by macrophage infiltration and ectopic lipid accumulation in liver and skeletal muscle, all of them associated with insulin resistance and cardiovascular disease [43].

#### Gc-induced osteoporosis

Chronic treatment with Gcs increase the risk of osteopenia, osteoporosis and aseptic osteonecrosis [36, 46]. Gcs decrease bone formation via inhibiting osteoblast proliferation and differentiation, reducing bone matrix protein synthesis in osteoblasts and increasing bone resorption via activating osteoclasts [46]. Mechanistically, both GR-mediated TA and TR are involved in Gc-induced osteoporosis. Gcs simultaneously stimulate the expression of RANKL (TNFSF11), a key factor for osteoclast differentiation/activation inhibiting osteoclast apoptosis, and reduce the expression of OPG (osteoprotegerin, TNFRSF11B), which promotes osteoclast apoptosis [46, 47]. Thus, Gcs increase RANKL/OPG ratio in bone, leading to osteoporosis. In addition, Gcs repress the expression of osteocalcin (OC), a key component of bone matrix produced by osteoblasts [48, 49]. Gcs also increase osteoclast activity by decreasing gastrointestinal Ca2+absorption and increasing urinary Ca2+excretion, which triggers an additional osteoclast-mediated bone resorption [46].

#### Gc-induced muscle waste

Glucocorticoids disrupt muscle homeostasis in multiple ways, by increasing catabolism, induction of ubiquitin-proteasomal pathway leading to proteolysis via transcriptional activation of E3 ligases atrogin-1 and MuRF1 [38]. The Gcs-mediated inhibition of IGF-1/PI3K/Akt pathway, the myostatin signaling and the NF-kB signaling also leads to inhibition of protein synthesis and to protein degradation [50]. In addition, glucocorticoids negatively affect muscle anabolism by inhibiting amino acid transport into muscles. mTOR, its inhibitor REDD1/DDIT4 and transcription factor KLF15 are involved in glucocorticoid-mediated repression of protein synthesis [50, 51]. In parallel, Gcs attenuate myogenic cell proliferation and differentiation, and reduce muscle mass [38].

#### Gc-mediated skin atrophy

Gc-induced skin atrophy is one of the most frequent side effects of topical and also systemic Gcs. It is characterized by a dramatic loss in skin thickness, increased fragility, tearing, bruising, permanent stretch marks (striae), and compromised skin barrier function along with delayed wound healing, followed by the increased risk for developing of secondary wounds and infections at the affected site [12, 52–55]. Gcs induce changes in all skin compartments, and appendages such as hair follicles and sebaceous glands. Typical changes in epidermis are hypoplasia, decreased number and size of keratinocytes, diminished stratum corneum. In dermis Gcs inhibit fibroblast proliferation, decrease collagen, elastin and other ECM proteins synthesis, reduce production of hyaluronic acid. Gcs also induce drastic atrophy of dermal adipose in mice after topical application and in patients after intradermal injections [56]. As discussed below, there are some parallels between mechanisms involved in skin atrophy and muscle waste, as the blockage of pro-proliferative anabolic mTOR/Akt signaling by Gcs plays central role in both skin and muscle steroid atrophy [37, 57].

### Approaches for the development of safer GR-targeted therapies

#### Modified classical glucocorticoids

Side effects associated with chronic high dose Gcs regimens made the search for safer GR-targeted therapies inevitable [58, 59]. Initially, the focus was on the modifications of the classical Gcs: delayed-release prednisone [60], fluticasone and budesonide with the specificity to lung tissues [58], targeted liposomal Gcs [61], nitrosteroids with the release of the low-dose nitric oxide and milder side effects than their parent compound prednisone [62], non-halogenated double-ester-type Gcs prednicarbate and 6-methyl-prednisolone aceponate that have reduced atrophogenic activity compared to conventional steroids. Another example is so-called soft drug approach by delivering Gcs that are active only at the site of action (e.g., in the lung or eye) but undergo a one-step predicted metabolism reducing the systemic exposure and limiting systemic side effects. Loteprednol etabonate and Ciclesonide have demonstrated efficacy and increased safety for the treatment of ophthalmic disorders and asthma respectively [63].

#### Development of dissociating selective GR activators/modulators (SEGRAM)

Alternative direction was the search for “dissociating” GR activators with improved therapeutic index. It started in late 1990s and was based on the concept of “dissociation” of GR TR and TA functions via ligands that do not activate GR dimerization and GR dimer-mediated TA linked to many atrophic and some metabolic adverse effects. A large number of research groups at pharmaceutical companies and academic institutions used both targeted synthesis of dissociating GR ligands and screening of chemical libraries to search for GR modulators that are currently called SEGRAM (selective GR agonists/modulators). Major tests used for detection and evaluation of TA and TR induced by SEGRAM as well as for evaluation of integral effects on inflammation and glucocorticoid-dependent side effects include *in vitro* molecular profiling of TR- and TA-associated genes, luciferase reporter assays – activation of GRE.Luciferase (for TA) and inhibition of NF-kB.Luciferase (for TR). The *in vitro* analysis is usually followed by the analysis of anti-inflammatory, metabolic, and atrophic effects *in vivo* (by ear edema and paw swelling assays, metabolic glucose test, Gcs-induced osteoporosis, skin atrophy and muscle waste models).

The first successful attempt at testing the activation/repression hypothesis was reported by Vayssiere *et al*. in 1997: the authors described several SEGRAM that separated *in vitro* TA and TR GR functions (RU24858, RU40066, and RU24782) [64]. Next generation of SEGRAM, compounds ZK245186 (Mapracorat/BOL-303242-X) and AL-438, demonstrated the affinity to GR close to Dex, the inhibition of the expression of inflammatory cytokines IL-1, IL-2, IL-8, prostaglandin-E2 and E-selectin as well as the anti-inflammatory therapeutic activity *in vivo* but exhibiting less undesirable effects than synthetic Gcs [54, 65–67]. Other synthetic SEGRAMs, compounds CpdX and CpdX-D3 related to Mapracorat, also demonstrated promising results in *in vivo* studies [68, 69].

SEGRAM PF-04171327 (Fosdagrocorat made by Pfizer) was in clinical trials for the treatment of rheumatoid arthritis [70, 71]. Fosdagrocorat showed higher therapeutic activity together with reduced side effects such as alteration of glucose metabolism and inhibition of bone formation [70, 71]. A number of potent, nonsteroidal, selective indazole ether based SEGRAM was developed by AstraZeneca for the inhaled treatment of respiratory diseases. AZD5423, AZD7594 and AZD9567 with high affinity to GR were in clinical trials for the therapy of chronic obstructive pulmonary disease (COPD) [72–74], asthma [75, 76] and rheumatoid arthritis [77], respectively. ZK216348 is a nonsteroidal SEGRAM with interesting properties which induces potent anti-inflammatory effects, but its capacity to transactivate is weaker compared to standard glucocorticoids [78, 79]. It was demonstrated that SEGRAM GSK866 by GlaxoSmithKline and its analogues with cysteine reactive warheads revealed stronger anti-inflammatory potential with less side effects in cutaneous and ocular inflammatory diseases, which is promising area for therapeutic intervention [80]. An interesting SEGRAM for topical use, LEO 134310 (LEO Pharma), is in clinical trials for psoriasis. It demonstrated minimal atrophic effects in skin and minimal systemic side effects as it was designed as a “dual-soft” GR ligand rapidly metabolized in liver and blood [81].

#### Natural compounds with SEGRAM properties

There have been extensive efforts to search for natural compounds (or their synthetic analogs) with SEGRAM properties, especially among the plant metabolites including terpens, terpenoids, polyphenols, and alkaloids that represent a rich source of bioactive compounds with beneficial health effects. Terpenes are the largest class of natural small-molecule metabolites mostly produced by conifers; they have poly-isoprene structure biosynthesized by condensation and modification of several isoprene (C_5_H_8_) units [82]. The sub-class of terpens are terpenoids that contain additional functional chemical groups and include well-known and widely used in traditional medicine cannabinoids, ginkolide and biobalide found in *Ginko biloba*, curcuminoids, ginsenosides found in *Panax ginseng*, and others. Notably, isoprenoid pathways are common for biosynthesis of steroids, including Gcs, and their major precursor cholesterol [83].

Importantly, some of plant secondary metabolites have typical steroid structure, and can bind various steroid hormone receptors [84]. They can act as agonists, antagonists, or modulators, and some can affect steroid metabolism, resulting in biological effects through altered endogenous steroid concentrations [84].

Significant number of plant-derived biomolecules have demonstrated capability to affect GR function which is usually assessed using cells expressing high levels of GR (for example, A549 cell line) and readout assays allowing for evaluation of GR TA/TR activity (for example, GRE.Luciferase and NF-kB.Luciferase reporter-based assays) [85, 86]. In some cases, more thorough investigations included analysis of effects on GR expression, major steps in GR activation: its phosphorylation (mostly at activating Ser211) and nuclear translocation; and GR ligand properties assessed by molecular docking or ligand-binding assays [87–94]. Recently, focused screens for natural mimetics of cortisol were performed utilizing genetically engineered sensor cells detecting GR nuclear translocation, which demonstrated that decursin from Dong quai (Angelica sinensis, commonly known as female ginseng), and L-limonene from peppermint oil induce GR translocation similar to cortisol [95, 96].

In addition, some other terpenes/terpenoids (astragaloside IV, avicin D, ginsenosides Re, Rg1 and compound K; boswellic acids, β-iscin; β-ionone) and other plant metabolites (berberine, extract from Salsola komarovii) were able to bind to GR. Moreover, many plant compounds including sstragaloside IV, avicin D, boswellic acids, berberine were able to induce significant nuclear translocation of GR ([85, 97–99] and Supplementary Table 1). However, in most cases these plant metabolites were not able to induce GR transactivation, and some compounds and plant extracts (curcumin, 4-hydroxyderricin and xanthoangelol, Peony Rubra Radix extract) inhibited GR TA ([100–102] and Supplementary Table 1). The exceptions are astragaloside IV, baicalein and endiandrin A that were able to activate GR-dependent genes/GRE. Luciferase reporter under certain experimental conditions [87, 97, 103].

At the same time, many discussed above compounds demonstrated SEGRAM or dissociating GR ligand activity. For example, boswellic acids suppressed GR TA and induced NF-kB TR via GR [98]; α-boswellic acid selectively inhibited CBG/Corticosteroid-binding globulin gene (marker for GR TR), but did not induce TAT/Tyrosine aminotransferase expression (key enzyme for glucocneogenesis and a marker for GR TA) [97]. Ginsenoside compound Rg1 and avicin D have also demonstrated GR TR but not TA activity [104].

The SEGRAM activity of these biomolecules was assessed not only in human cell lines, but in some cases *in vivo* in zebrafish larvae model and in rodents ([87, 95, 98, 100, 102–106] and [99, 101, 107, 108], respectively).

Many of these natural compounds with SEGRAM properties have anti-inflammatory, antimicrobial, and anti-cancer activity [85, 109, 110], and several SEGRAMs listed above are active components of traditional medicinal herbs. For example, preparations from woody vines rich in boswellic acids are used in China to treat rheumatoid arthritis and inflammation; decursin is a component of Dong quai, traditional Chinese medicinal herb; Ginseng is the traditional medicinal herb and one of the top-selling herbal supplement worldwide [111]). However, the clinical use of many other compounds is limited because of narrow therapeutic window and diverse toxic effects (triptolide [108, 112]), or low bioavailability (astragaloside IV [113]).

One of the interesting examples of synthetic analogs of natural SEGRAM is Compound A (CpdA), a stable analog of the hydroxyphenyl aziridine precursor found in the Namibian shrub Salsola tuberculatiformis Botschantzev [5]. Others and we showed that CpdA has high affinity to GR. In different cell types CpdA induced modest GR nuclear translocation but did not induce GR dimerization and GR phosphorylation at Ser211, critical for GR TA activity [5]. Consequently, CpdA did not significantly affect or even inhibited constitutive and Gcs-induced activation of endogenous genes. In contrast, CpdA and classical Gcs have remarkably similar TR profiles, suppressing the activity of many pro-proliferative and anti-apoptotic TFs including NF-κB, AP-1, Ets-1, Elk-1, SRF, NFATc [114]. Importantly, CpdA demonstrated strong anti-inflammatory activity in multiple models of inflammatory diseases (collagen-induced arthritis, experimental autoimmune neuritis and encephalomyelitis, type 1 diabetes, Th2-driven mouse asthma model), as well as strong anti-cancer effects with minimal adverse effects as measured by glucose metabolism and skin atrophy [7, 11, 115–122].

#### Shift of paradigm: from dissociating GR ligands to partial GR agonists

Overall, during last 20 years the major efforts by pharmaceutical companies and academia to make GR-targeted therapies safer have been focused on the development of GR agonists/modulators (SEGRAM) that can dissociate TR and TA GR functions. However, despite of some progress, and many interesting findings related to both synthetic and natural compounds, especially in *in vitro* studies and in some animal models, the attempts to generate/discover truly dissociating GR ligands/modulators had limited success, and only few SEGRAMs have reached clinical trials. Moreover, even leading SEGRAMs such as Fosdagrocorat (Pfizer) developed for the treatment of rheumatoid arthritis and Mapracorat that was in clinical trials for atopic dermatitis (Bayer) and ocular allergic conjunctivitis (by Bausch & Lomb) have never been marketed, and their further development was put on hold by the companies. Similarly, none of the natural compounds with SEGRAM properties that have been used in traditional medicine for a long time, have been approved as prescription drugs, and they are sold currently only as dietary supplements.

The difficulties with SEGRAM development reflect the significant limitations of the TR/TA dissociation concept, first of all because the analysis of Gcs molecular signatures in different tissues including liver, brain, skin revealed that side effects as well as full therapeutic activity of Gcs require both TA and TR [123]. For example, Gcs upregulate anti-inflammatory genes glucocorticoid-induced leucine zipper (GILZ) and dual specificity phosphatase (DUSP1), and cell cycle inhibitors p21 and p27 [124–126]. On the other hand, Gcs inhibit OPG (osteoprotegerin), one of the key anti-osteoporotic genes in bone [7, 12, 123, 127, 128]. In case of osteoporosis, up-regulation of proapoptotic genes contributes to Gc-induced apoptosis in osteocytes, and inhibition of OPG together with RANKL up-regulation lead to enhanced osteoclastogenesis [129]. Up-regulation of Plasminogen Activator Inhibitor 1 (PAI-1) by Gcs in the patients with inflammation increased the risk of venous thromboembolism via inhibition of the breakdown of blood clots [130, 131].

The other problems with the search/design of SEGRAM are the lack of three-dimensional structures of full-length GR that preclude efficient molecular modeling and docking studies; need of assays with high predictive power for screening of SEGRAM anti-inflammatory and specific adverse effects in human cells *in vitro*; the lack of translatability from animal models to human patients [123].

Currently, the approach for SEGRAM design shifted from dissociating GR ligands towards GR partial agonists following the strategy successfully used for the development of selective ER modulators (SERMs). Partial non-steroidal GR agonists AZD7495 and AZD9567 developed by AstraZeneca retained full capability to induce GR TR (assessed by inhibition of LPS induced TNFα release in human blood cells), but did not activate specific genes involved in osteoporosis (induction of osteoprotegerin, OPG, in human osteoblasts) and glucose metabolism (assessed in human hepatocytes by activation of tyrosine aminotransferase, TAT). AZD9567 demonstrated excellent anti-inflammatory activity in animal experiments, and is currently in clinical trials for rheumatoid arthritis and type 2 diabetes in comparison with prednisolone [77].

### Combination therapy approach to increase therapeutic index of Glucocorticoids

As discussed above, it became increasingly clear that it is nearly impossible to design/select truly dissociating GR ligands that can reduce multiple side effects while retaining the therapeutic activity of classical Gcs. Much more feasible strategy for safer GR-targeted therapies is to focus on the alleviation of specific side effects taking into consideration cell/tissue-specific GR/Gcs molecular signatures. The examples below illustrate the successful attempts (both empirical and via multi-step screening) to increase the therapeutic index of Gcs using combination of Gcs with some other drugs.

#### Combinational therapies to increase therapeutic effects of Gcs

Glucocorticoid monotherapy is adequate for some inflammatory diseases, which require low-dose treatments. However, for more severe inflammatory and autoimmune disorders as well as blood cancer, Gcs are combined with other drugs.

Interestingly, some combination therapies increased therapeutic effects of Gcs without exacerbation of their adverse effects. For example, combination of prednisolone with anti-thrombotic drug dipyridamole resulted in an increased anti-inflammatory effect of prednisolone through selective amplification of Gcs-mediated anti-inflammatory signaling [132]. Another recent work demonstrated that GR binding to GREs in *β-arrestin 1* and *2* and modulates their expression and alters G-protein coupled receptor (GPCR) signaling which may have beneficial implications in combination therapy using corticosteroids and GPCR-based drugs in the treatment of asthma and COPD [133]. Further, it was discovered that rapamycin sensitized multiple myeloma, leukemia and lymphoma cells to Dex-induced apoptosis, increasing its anti-lymphoma effects [134, 135]. Unfortunately the promising approach to induce “dissociation” of GR TR from TA, and to increase therapeutic Gcs index by using Dex together with SEGRAM CpdA, failed [136].

#### Combinational therapies to decrease atrophic effects of Gcs

There are also findings (sometime by serendipity, but frequently by design) related to the decrease of atrophic and metabolic adverse effects when Gcs were combined with other drugs. For example, it was shown that glycyrrhizic acid from Licorice root, dipyridamole (mentioned above), and parathyroid hormone (PTH) were able to attenuate the effects of Gcs on bone loss in rodents [1, 108, 117, 118]. Japanese studies demonstrated that branched amino acids have protective effect against steroid-induced muscle dystrophy in animal models and also in patients with rheumatic disorders due to the blockage of catabolic processes in muscle [119, 123].

There are also important findings related to the combination of Gcs with other steroid receptor/nuclear receptor ligands. Vitamin D_3_ and its analogues diminish anti-atrophic effects of glucocorticoids in muscle and bone [137–141]. As there is an overlap in enzymatic cascades involved in the synthesis of Gcs and Vitamin D_3_, some Vitamin D metabolites could also act as potential modulators of GR activity via effects on Gcs synthesis/metabolism [142–144].

It is known that Gcs bind not only GR but also mineralocorticoid receptor (MR), and that inhibitors of MR, such as spironolactone are considered for co-treatment with glucocorticoids to reduce some adverse effects, such as skin atrophy [145]. The inhibitors of 11βHSD1 that plays a key role in regulation of glucocorticoids in tissues, were also considered for the reduction of glucocorticoid side effects (for example, Glycyrrhetinic acid, KR-67607, AZD4017) [146–149].

#### Search for tissue protectors to spare tissues from steroid atrophy

The strategically different systematic approach for combining of Gcs with tissue protectors requires the identification of the Gcs-induced genes causatively implicated in specific Gcs adverse effects followed by targeted inhibition of their expression. We validated this approach, using skin atrophy as a model, via analysis of GR molecular signature in skin, identification and validation of potential atrophogenes and search (via repurposing approach) for small molecule drugs that could inhibit atrophogene expression in steroid-treated skin. Later this approach was extended towards osteoporosis model. We used the model of Gc-induced osteoporosis in mice and demonstrated the potency of PI3K/Akt/mTOR modulators to diminish bone resorption induced by Dex [150].

### Identification of key atrophogenes involved in atrophic effects of glucocorticoids

Bioinformatics analysis of GR target genes upregulated both in human and mouse skin topically treated by Gcs, revealed several dozens of common upregulated differentially expressed genes (DEGs, GEO accession numbers: GSE120783, GSE59151 [35, 42]). Several of these DEGs appeared to be negative regulators of major anabolic mTOR/Akt signaling pathway, including REDD1 (regulated in development and DNA damage 1)/DDIT4 (DNA damage induced transcript 4) and FKBP5 (FK506-binding protein 51) [42, 61]. Both of these genes are GR targets [151–154]. The major function of REDD1 is negative regulation of mTORC1, while FKBP51 serves as a molecular chaperone for multiple clients. However, they both play the important role in negative regulation of mTOR/Akt signaling via control of Akt dephosphorylation: FKBP51 -at Ser473 [155], and REDD1 -at Thr308 [156].

We demonstrated that REDD1 and FKBP51 indeed act as atrophogenes in skin. Both REDD1 knockout (KO) and FKBP5 KO animals were more resistant to Gcs-induced skin hypoplasia than wild type mice: the lack of REDD1 or FKBP51 expression safeguarded all skin compartments (epidermis, dermis and dermal adipose) and protected CD34+ follicular epithelial stem cells from negative steroid effects [42, 61].

REDD1 role in the regulation of muscle metabolism and shift of protein synthesis/degradation balance towards catabolism during treatment with Gcs is well recognized [123]. Moreover, the finding that REDD1 KO animals appeared to be more resistant to Gcs-induced muscle waste provided direct experimental evidence that REDD1 plays also a causative role in steroid-induced muscle atrophy [56]. The role of FKBP51 in muscle atrophy remains to be investigated.

### GR and PI3K/Akt/mTOR crosstalk is involved in glucocorticoid-induced atrophy in skin and bone

**Figure 1:**
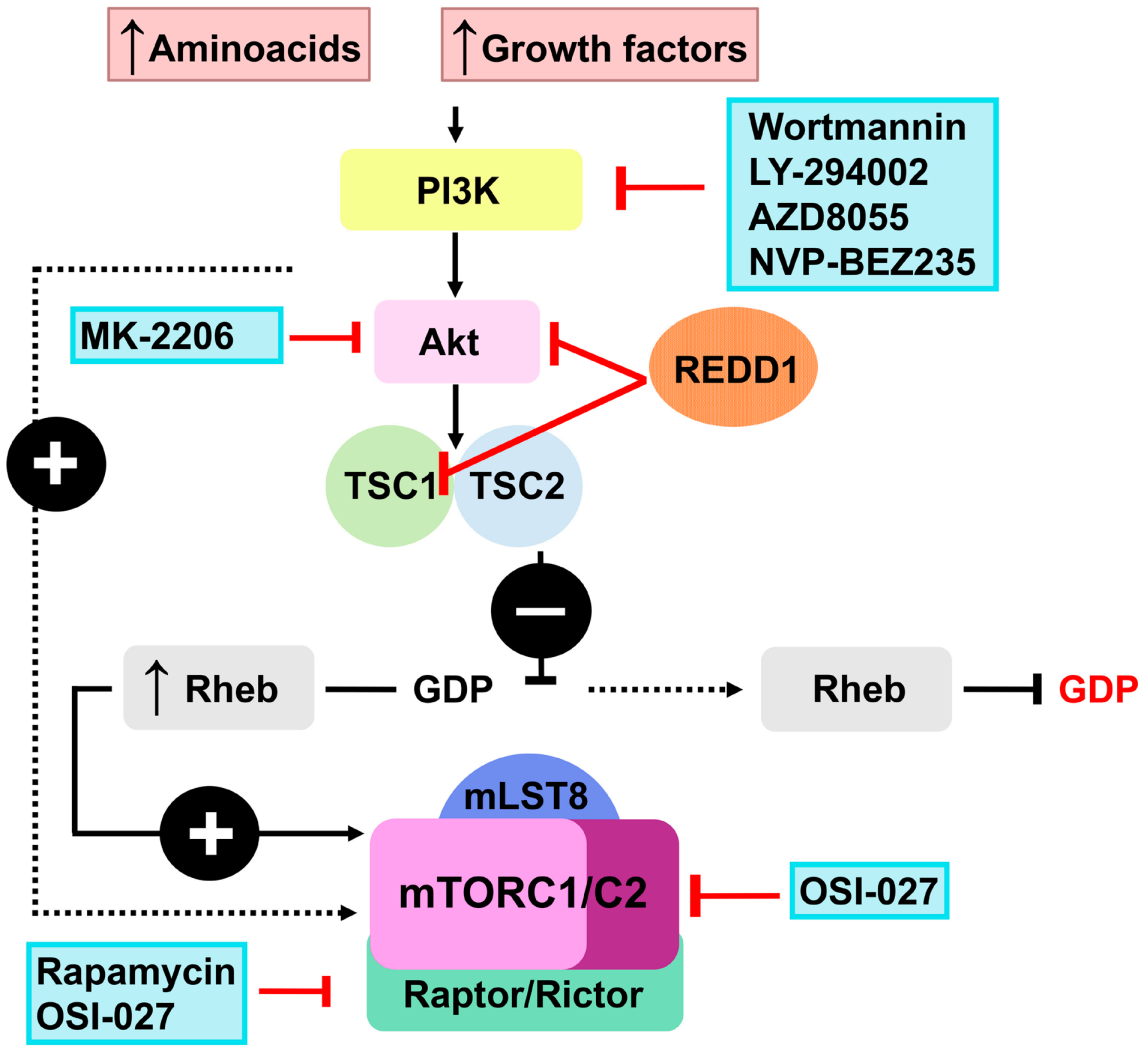
Putative REDD1 inhibitors from the PI3K/Akt/mTOR modulators class.

The computational screening of LINCS database of ~ 20,000 transcriptional signatures induced by FDA-approved and experimental drugs (http://lincsproject.org/LINCS/) identified a significant number of putative inhibitors of REDD1 expression among pharmacological class of PI3K/Akt/mTOR inhibitors [150, 154, 157] including classical experimental inhibitors such as LY294002 and Wortmannin (WM), and drugs in clinic/clinical trials such as Rapamycin (Rapa) and AZD8055 (Figure 1). This was completely unexpected as PI3K/Akt/mTOR inhibitors block major pro-proliferative signaling in cells.

The experimental validation of multiple repurposing candidates from this pharmacological class, demonstrated that they indeed inhibited basal and Gcs-induced REDD1 expression in keratinocytes and blood cancer cells *in vitro* and in mouse skin *in vivo* (Table 1, [116, 124, 133]). Most of tested PI3K/Akt/mTOR inhibitors also blocked Gcs-induced FKBP51 expression [133]. In addition, we recently showed that Rapamycin inhibited both basal and Dex-induced REDD1 expression in osteocytes (Supplementary Figure 1). This is an important finding even though the role of REDD1 in Gcs-induced osteoporosis remains to be investigated.

**Table 1:** Effects of PI3K/Akt/mTOR inhibitors on REDD1 basal and inducible expression in HaCaT keratinocytes

Compound	Major Targets	REDD1 basal RNA/protein		REDD1 Gc-induced RNA/protein		FKBP51 induced RNA/protein	
Rapamycin	mTORC1	+	+	+	+	+	+
OSI-027	mTORC1/2	+	+	+	+	N/A	N/A
Wortmannin	PI3K	+	+	+	+	+	+
LY-294002	PI3K	+	+	+	+	+	+
NVP-BEZ235	PI3K/mTOR	N/A	+	N/A	+	+	+
AZD8055	PI3K/mTOR	+	+	+	+	+	+
MK-2206	Akt1/2/3	N/A	+	N/A	+	+	+

HaCaT keratinocytes were pretreated with REDD1 inhibitors (1–10 uM) for hrs, and treated with FA (1 uM) for 6–24 hrs. The expression of REDD1 was determined by Western blotting and Q-PCR and normalized to Rpl27 expression. References: [150, 154]. Abbreviation: N/A: not assessed.

Using two different models of steroid atrophy – skin atrophy and osteoporosis, we validated protective effects of PI3K inhibitors when they and Gcs were delivered either topically or systemically. Indeed, topical application of Rapamycin or LY294002 together with glucocorticoid fluocinolone acetonide (FA) protected mice against FA-induced proliferative block and its atrophic effects in epidermis, dermis and dermal adipose [124, 133]. We also found that systemic co-administration of LY294002 or Rapamycin with Dex protected skin against Dex-induced atrophy. These PI3K inhibitors also normalized RANKL/OPG ratio and collagen expression in bone indicating a reduction of Dex-induced osteoporosis [116].

Further, we demonstrated that combination of Gcs with PI3K inhibitors did not affect anti-inflammatory activity of Gcs in croton oil ear edema test [124, 133]. Moreover, Rapamycin and LY294002 enhanced anti-lymphoma effects of Dex in human lymphoma xenograft model, and the therapeutic effects of PI3K inhibitor + Dex combinations ranged from cooperative to synergistic compared to single treatment [150].

One of the most intriguing findings in this work was the ability of several PI3K/Akt/mTOR inhibitors (including Rapamycin, LY294002, Wortmannin, AZD8055) to modify GR function, shifting GR activity towards therapeutically important TR in keratinocytes and lymphoid cells. This was assessed by activation of GRE.Luciferase (for TA) and inhibition of NF-kB Luciferase (for TR), and by global changes in Gcs transcriptome validated by Q-PCR of GR target genes (Table 2, [150, 154, 157]). Interestingly, some PI3K/Akt/mTOR inhibitors negatively affected GR phosphorylation at critical for TA Ser211; GR nuclear translocation; GR loading on REDD1/FKBP51 gene promoters and the expression of other DEGs upregulated by Gcs [150, 154, 157]. At the same time, PI3K/Akt/mTOR inhibitors increased negative effect of Gcs on central pro-inflammatory/pro-proliferative factor NF-kB and increased down-regulation of cell cycle (Cyclins and Cdks), and pro-inflammatory genes (interleukins, chemokines, cytokines) by Gcs [150, 154, 157].

**Table 2:** Effect of PI3K/Akt/mTOR inhibitors on GR function

Compound	Major Targets	Gene activation by glucocorticoids	Gene inhibition by glucocorticoids
Rapamycin	mTORC1	Blunted (validated array)	Exaggerated (validated array)
OSI-027	mTORC1/2	N/A	N/A
Wortmannin	PI3K	Blunted	Exaggerated
LY-294002	PI3K	Blunted (validated array)	Exaggerated (validated array)
NVP-BEZ235	PI3K/mTOR	N/A	N/A
AZD8055	PI3K/mTOR	Blunted	Exaggerated
MK-2206	Akt1/2/3	N/A	N/A

HaCaT keratinocytes were pretreated with REDD1 inhibitors (1–10 uM) for hrs, and treated with FA (1 uM) for 6–24 hrs. The level of gene activation/repression was determined by Luciferase reporter assay. References: [137, 140]. Abbreviation: N/A: not assessed.

Overall, these results provide a proof of principle for using drug repurposing approach to target specific Gcs-induced atrophogenes in selected tissues including skin, muscle and bone.

## CONCLUSIONS

Gcs were introduced in clinic more than 70 years ago, but still remain among most widely used drugs for the treatment of autoimmune and inflammatory diseases and blood cancer. Unfortunately, Gcs are also notorious for multiple metabolic, atrophic and other adverse effects. The extensive efforts to reduce Gcs adverse effects and improve their therapeutic index were initially focused on the development of dissociating GR ligands capable to down-regulate pro-inflammatory genes but lacking Gcs transactivation potential linked to their adverse effects. Overall, the attempts to generate truly dissociating GR activators/modulators (SEGRAM) had limited success, and recently, the approach for SEGRAM design shifted towards GR partial agonists following the strategy successfully used for the development of selective modulators of estrogen receptor. Another promising strategy to reduce Gcs tissue- specific adverse effects is to use them in combination with tissue protectors, which seems to be especially beneficial for prevention/alleviation of Gcs atrophic effects in skin, muscle and bone. We discussed here in detail a systematic approach to identify the GR target genes causatively involved in atrophic effects of Gcs (atrophogenes) and the search for candidate small molecule drugs (anti-atrophogenes) that could inhibit their expression. Using steroid-induced skin atrophy as a model, we identified and validated REDD1 and FKBP51 as genes central for atrophy in skin and discovered that many PI3K/Akt/mTOR inhibitors can strongly down-regulate the expression of these atrophogenes in skin, lymphoma cells, and in osteocytes. We proved that PI3K/Akt/mTOR inhibitors can protect skin against atrophy and extended this observation using osteoporosis model. The combination of Gcs with PI3K inhibitors also appeared to be exceptionally promising as it did not affect anti-inflammatory and enhanced anti-lymphoma activity of Gcs in human xenograft models. Overall, these new approaches suggest feasibility of a really improved safer GR-targeted therapies so much needed for millions of patients with inflammatory, autoimmune diseases and blood cancer.

## SUPPLEMENTARY MATERIALS





## Abbreviations

CpdA: Compound A
DEG: differentially expressed genes
Dex: dexamethasone
DDIT4: DNA damage induced transcript 4
FA: fluocinolone acetonide
FKBP5: FK506-binding protein 51
11βHSD1: 11β-hydroxysteroid dehydrogenase type 1
Gc(s): glucocorticoid(s)
GILZ: glucocorticoid-induced leucine zipper
GR: glucocorticoid receptor
GRE: glucocorticoid responsive elements
LINCS: library of integrated network-based cellular signatures
mTOR: mammalian target of rapamycin
RA: rheumatoid arthritis
REDD1: regulated in development and DNA damage response 1
SEGRAM: selective glucocorticoid receptor agonist/modulator
TA: transactivation
TAT: tyrosine aminotransferase
TF: transcription factor
TR: transrepression
