# Supplementary material for: The long winding road to the safer glucocorticoid receptor (GR) targeting therapies
Source: Oncotarget. 2022 Feb 18;13:408–24. doi: 10.18632/oncotarget.28191 (PMC8858080; doi:10.18632/oncotarget.28191)
Supplement: Supplementary file 1 [file oncotarget-13-28191-s001.pdf]

The long winding road to the safer glucocorticoid receptor (GR) targeting therapies

SUPPLEMENTARY MATERIALS

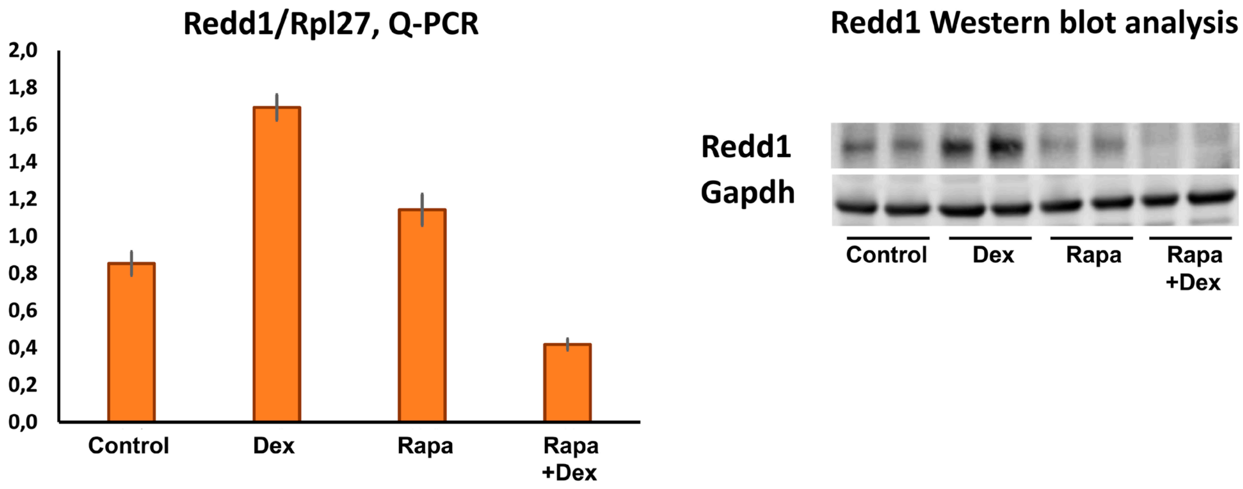

Supplementary Figure 1: Rapamycin prevents induction of atrophogene REDD1 by Dex in murine osteocytes. MC3T3 murine preosteoblast cells were grown in MEM alpha medium supplemented with 10% FBS till confluency, and then were placed in the differentiating medium for one week. Following the differentiation period, the cells were pre-treated with Rapamycin (Rapa, 1 uM) or vehicle (Control) for 6 hrs, and then treated with Dex or vehicle for 24 h. The expression of REDD1 was determined by Western blotting and Q-PCR and normalized to Rpl27 expression. The results of one representative experiment (two biological repeats/group) are presented. Differentiating medium: MEM alpha medium with 10% FBS, supplemented with 30 mM Beta-glycerophospate disodium salt hydrate (Sigma G-9422), and 50 mM L –ascorbic acid 2-phosphate sesquimagnesium salt hydrate (Sigma A8960).

Supplementary Table 1: Natural compounds with SEGRAM properties. See Supplementary Table 1

SUPPLEMENTARY REFERENCES

1. Mamedova RP, Isaev MI. Triterpenoids from Astragalus plants. Chem Nat Compd. 2004; 40:303–57. https://doi.org/10.1023/B:CONC.0000048246.16075.62.
2. Liu HS, Shi HL, Huang F, Peterson KE, Wu H, Lan YY, Zhang BB, He YX, Woods T, Du M, Wu XJ, Wang ZT. Astragaloside IV inhibits microglia activation via glucocorticoid receptor mediated signaling pathway. Sci Rep. 2016; 6:19137. https://doi.org/10.1038/srep19137. [PubMed]
3. Jayatilake GS, Freeberg DR, Liu Z, Richheimer SL, Blake Nieto ME, Bailey DT, Haridas V, Gutterman JU. Isolation and structures of avicins D and G: *in vitro* tumor-inhibitory saponins derived from Acacia victoriae. J Nat Prod. 2003; 66:779–83. https://doi.org/10.1021/np020400v. [PubMed]
4. Haridas V, Xu ZX, Kitchen D, Jiang A, Michels P, Gutterman JU. The anticancer plant triterpenoid, avicin D, regulates glucocorticoid receptor signaling: implications for cellular metabolism. PLoS One. 2011; 6:e28037. https://doi.org/10.1371/journal.pone.0028037. [PubMed]
5. Gao Z, Huang K, Yang X, Xu H. Free radical scavenging and antioxidant activities of flavonoids extracted from the radix of Scutellaria baicalensis Georgi. Biochim Biophys Acta. 1999; 1472:643–50. https://doi.org/10.1016/S0304-4165(99)00152-X. [PubMed]
6. Austin JR, Kirkpatrick BJ, Rodríguez RR, Johnson ME, Lantvit DD, Burdette JE. Baicalein Is a Phytohormone that Signals Through the Progesterone and Glucocorticoid Receptors. Horm Cancer. 2020; 11:97–110. https://doi.org/10.1007/s12672-020-00382-6. [PubMed]
7. Neag MA, Mocan A, Echeverría J, Pop RM, Bocsan CI, Crişan G, Buzoianu AD. Berberine: Botanical Occurrence, Traditional Uses, Extraction Methods, and Relevance in Cardiovascular, Metabolic, Hepatic, and Renal Disorders. Front Pharmacol. 2018; 9:557. https://doi.org/10.3389/fphar.2018.00557. [PubMed]
8. Liang Y, Zhang T, Zhao J, Li C, Zou H, Li F, Zhang J, Ren L. Glucocorticoid receptor-mediated alleviation of inflammation by berberine: *in vitro*, in silico and *in vivo* investigations. Food Funct. 2021; 12:11974–86. https://doi.org/10.1039/D1FO01612A. [PubMed]
9. Al-Harrasi A, Rehman NU, Khan AL, Al-Broumi M, Al-Amri I, Hussain J, Hussain H, Csuk R. Chemical, molecular and structural studies of Boswellia species: β-Boswellic Aldehyde and 3-epi-11β-Dihydroxy BA as precursors in biosynthesis of boswellic acids. PLoS One. 2018; 13:e0198666. https://doi.org/10.1371/journal.pone.0198666. [PubMed]
10. Karra AG, Tziortziou M, Kylindri P, Georgatza D, Gorgogietas VA, Makiou A, Krokida A, Tsialtas I, Kalousi FD, Papadopoulos GE, Papadopoulou KK, Psarra AG. Boswellic acids and their derivatives as potent regulators of glucocorticoid receptor actions. Arch Biochem Biophys. 2020; 695:108656. https://doi.org/10.1016/j.abb.2020.108656. [PubMed]
11. Kupchan SM, Court WA, Dailey RG Jr, Gilmore CJ, Bryan RF. Triptolide and tripdiolide, novel antileukemic diterpenoid triepoxides from Tripterygium wilfordii. J Am Chem Soc. 1972; 94:7194–95. https://doi.org/10.1021/ja00775a078. [PubMed]
12. Yang M, Shen JK, Huang J, Du HP, Ma QL, Jin J. Interleukin-6-independent expression of glucocorticoid receptor is upregulated by triptolide in multiple myeloma. Leuk Lymphoma. 2009; 50:802–08. https://doi.org/10.1080/10428190902801838. [PubMed]
13. Wu J, Chen G, Xu X, Huo X, Wu S, Wu Z, Gao H. Seven new cassane furanoditerpenes from the seeds of Caesalpinia minax. Fitoterapia. 2014; 92:168–76. https://doi.org/10.1016/j.fitote.2013.11.002. [PubMed]
14. Xiang G, Fan M, Ma Y, Wang M, Gao J, Chen J, Li X, Xue W, Wang Y, Gao H, Shen Y, Xu Q. Anti-inflammatory actions of Caesalpinin M2 in experimental colitis as a selective glucocoricoid receptor modulator. Biochem Pharmacol. 2018; 150:150–59. https://doi.org/10.1016/j.bcp.2018.02.003. [PubMed]
15. Soleimani V, Sahebkar A, Hosseinzadeh H. Turmeric (Curcuma longa) and its major constituent (curcumin) as nontoxic and safe substances: review. Phytother Res. 2018; 32:985–95. https://doi.org/10.1002/ptr.6054. [PubMed]
16. Aoyagi S, Archer TK. Differential glucocorticoid receptor-mediated transcription mechanisms. J Biol Chem. 2011; 286:4610–19. https://doi.org/10.1074/jbc.M110.195040. [PubMed]
17. Sarker SD, Nahar L. Natural medicine: the genus Angelica. Curr Med Chem. 2004; 11:1479–500. https://doi.org/10.2174/0929867043365189. [PubMed]
18. Kang C, Kim S, Lee E, Ryu J, Lee M, Kwon Y. Genetically Encoded Sensor Cells for the Screening of Glucocorticoid Receptor (GR) Effectors in Herbal Extracts. Biosensors (Basel). 2021; 11:341. https://doi.org/10.3390/bios11090341. [PubMed]
19. Sergeant CA, Africander D, Swart P, Swart AC. Sutherlandia frutescens modulates adrenal hormone biosynthesis, acts as a selective glucocorticoid receptor agonist (SEGRA) and displays anti-mineralocorticoid properties. J Ethnopharmacol. 2017; 202:290–301. https://doi.org/10.1016/j.jep.2017.03.019. [PubMed]
20. Gallelli L, Cione E, Wang T, Zhang L. Glucocorticoid-Like Activity of Escin: A New Mechanism for an Old Drug. Drug Des Devel Ther. 2021; 15:699–704. https://doi.org/10.2147/DDDT.S297501. [PubMed]
21. Zhao SQ, Xu SQ, Cheng J, Cao XL, Zhang Y, Zhou WP, Huang YJ, Wang J, Hu XM. Anti-inflammatory effect of external use of escin on cutaneous inflammation: possible involvement of glucocorticoids receptor. Chin J Nat Med. 2018; 16:105–12. https://doi.org/10.1016/S1875-5364(18)30036-0. [PubMed]
22. Xin W, Zhang L, Fan H, Jiang N, Wang T, Fu F. Escin attenuates acute lung injury induced by endotoxin in mice. Eur J Pharm Sci. 2011; 42:73–80. https://doi.org/10.1016/j.ejps.2010.10.008. [PubMed]

23. Kuntzsch D, Bergann T, Dames P, Fromm A, Fromm M, Davis RA, Melzig MF, Schulzke JD. The plant-derived glucocorticoid receptor agonist Endiandrin A acts as co-stimulator of colonic epithelial sodium channels (ENaC) via SGK-1 and MAPKs. PLoS One. 2012; 7:e49426. https://doi.org/10.1371/journal.pone.0049426. [PubMed]
24. Leung KW, Leung FP, Huang Y, Mak NK, Wong RN. Non-genomic effects of ginsenoside-Re in endothelial cells via glucocorticoid receptor. FEBS Lett. 2007; 581:2423–28. https://doi.org/10.1016/j.febslet.2007.04.055. [PubMed]
25. Li Y. [Relationship between glucocorticoid receptor and deficiency syndrome and the regulation of traditional Chinese medicine]. [Article in Chinese]. J Chin Integr Med. 2004; 2:172–74. [PubMed]
26. He M, Halima M, Xie Y, Schaaf MJ, Meijer AH, Wang M. Ginsenoside Rg1 Acts as a Selective Glucocorticoid Receptor Agonist with Anti-Inflammatory Action without Affecting Tissue Regeneration in Zebrafish Larvae. Cells. 2020; 9:E1107. https://doi.org/10.3390/cells9051107. [PubMed]
27. Yoshioka Y, Samukawa Y, Yamashita Y, Ashida H. 4-Hydroxyderricin and xanthoangelol isolated from Angelica keiskei prevent dexamethasone-induced muscle loss. Food Funct. 2020; 11:5498–512. https://doi.org/10.1039/D0FO00720J. [PubMed]
28. Wu X, Wu J, Xia S, Li B, Dong J. Icaritin opposes the development of social aversion after defeat stress via increases of GR mRNA and BDNF mRNA in mice. Behav Brain Res. 2013; 256:602–08. https://doi.org/10.1016/j.bbr.2013.09.034. [PubMed]
29. Choi D, Kang W, Park S, Son B, Park T. β-Ionone Attenuates Dexamethasone-Induced Suppression of Collagen and Hyaluronic Acid Synthesis in Human Dermal Fibroblasts. Biomolecules. 2021; 11:619. https://doi.org/10.3390/biom11050619. [PubMed]
30. Seo JH, Jin MH, Chang YH. Anti-inflammatory effect of Salsola komarovii extract with dissociated glucocorticoid activity. BMC Complement Med Ther. 2020; 20:176. https://doi.org/10.1186/s12906-020-02979-4. [PubMed]
31. Juan YC, Chang CC, Tsai WJ, Lin YL, Hsu YS, Liu HK. Pharmacological evaluation of insulin mimetic novel suppressors of PEPCK gene transcription from Paeoniae Rubra Radix. J Ethnopharmacol. 2011; 137:592–600. https://doi.org/10.1016/j.jep.2011.06.007. [PubMed]
32. Lesovaya E, Yemelyanov A, Swart AC, Swart P, Haegeman G, Budunova I. Discovery of Compound A—a selective activator of the glucocorticoid receptor with anti-inflammatory and anti-cancer activity. Oncotarget. 2015; 6:30730–44. https://doi.org/10.18632/oncotarget.5078. [PubMed]
