# Supplementary material for: The long winding road to the safer glucocorticoid receptor (GR) targeting therapies
Source: Oncotarget. 2022 Feb 18;13:408–24. doi: 10.18632/oncotarget.28191 (PMC8858080; doi:10.18632/oncotarget.28191)
Supplement: Supplementary file 2 [file oncotarget-13-28191-s002.docx]

**Supplementary Table 1. Natural compounds with SEGRAM properties**

| **Biomolecule/plant source** | **Chemical type** | **TA** | **TR** | **GR nuclear translocation** | **GR expression** | **GR binding** | **Chemical structure** | **Reference** |
| --- | --- | --- | --- | --- | --- | --- | --- | --- |
| **Astragaloside IV**  **From *Astragalus genus*** | terpen | activates the GR-mediated signaling | Inhibits  NF-kB | + |  | + | 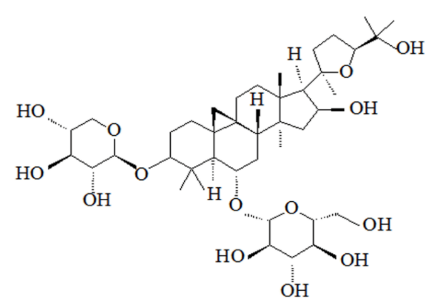 | [1, 2] |
| **Avicin D**  **From *Acacia victoriae*** | terpen |  |  | + |  | binds only to the GR  in antagonistic conformation | 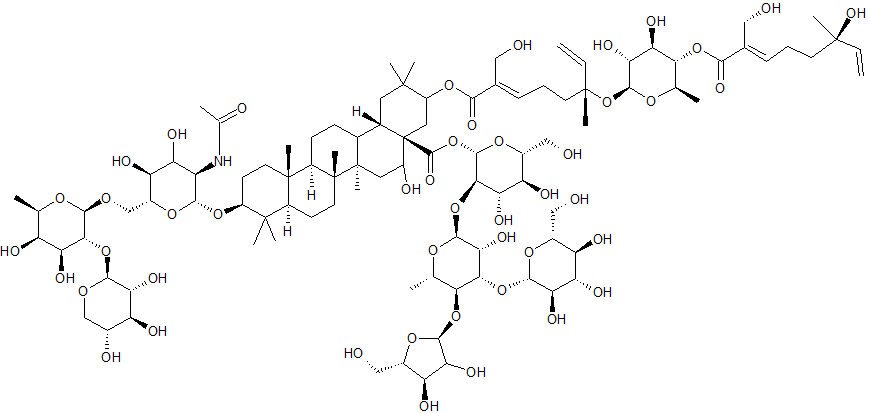 | [3, 4] |
| **Baicalein** **From Scutellaria baicalensis*Georgi*** | flavonoid | GR agonist activity |  |  |  |  | 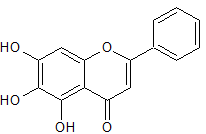 | [5, 6] |
| **Berberine**  **From Berberis genus** | alkaloid |  |  | + |  | + | 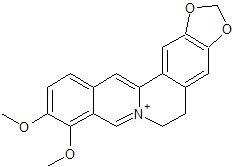 | [7, 8] |
| **Boswellic acids**  **From *Boswellia* genus** | terpenoids | no | Modulates TR | + |  | + | 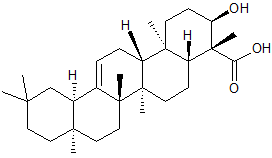 | [8–10] |
| **Triptolide**  **From *Tripterygium wilfordii*** | terpen |  |  |  | increases |  | 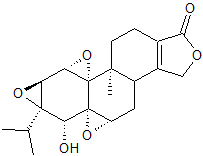 | [11, 12] |
| **Caesalpinin M2**  **From *Caesalpinia minax Hance*** | terpen | no | inhibitsNF-κB |  |  | + | 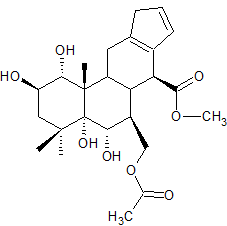 | [13, 14] |
| **Curcumin**  **From Curcuma longa** | polyphenol | Inhibits, but does not affect GR binding to GREs |  |  |  |  | 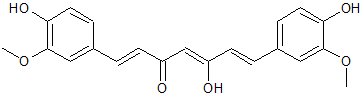 | [15, 16] |
| **Decursin**  **From *Angelica* genus** | pyranocoumarin | selective GR agonist activity |  |  |  |  | 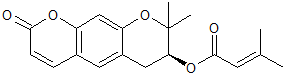 | [17, 18] |
| ***Sutherlandia frutescens* extract** | Mixture of several molecules with major bioactive components yet to be identified |  | + |  |  |  | Mixture of several molecules with major bioactive components yet to be identified | [19] |
| **Escin beta**  **From *Aesculus hippocastanum*** | terpene (saponin) |  |  |  | increases | + | 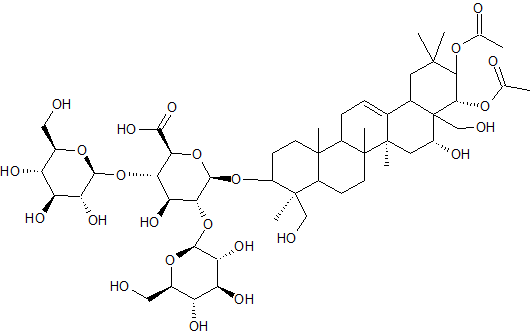 | [20–22] |
| **Endiandrin A**  **From Endiandra anthropophagorum*Domin*** | cyclobutane lignan | Slightly increases TA |  |  | increases |  | 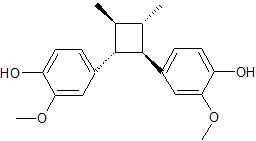 | [23] |
| **Ginsenosides**  **From *Panax ginseng*** | steroid glycosides and triterpene saponins | No (ginsenoside compound Rg1) | Decreases TR  (Ginsenoside compound Rg1) |  |  | + | 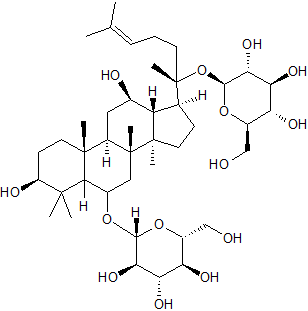 | [24–26] |
| **4-Hydroxyderricin and xanthoangelol**  **From *Angelica keiskei*** | prenylated chalcones | Decreases TA |  | decreases nuclear translocation thus decreasing muscle waste |  |  | 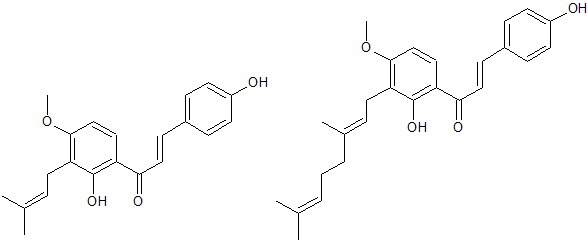 | [27] |
| **Icaritin**  **From Herba Epimedii** | flavonoids |  |  | _+_ |  |  | 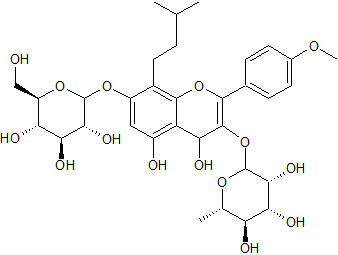 | [28] |
| **β-ionone**  **widely present in flowers, fruits, and vegetables such as carrots** | terpene |  | GR partial antagonist (Decreases negative effect on collagen genes) |  |  | + | 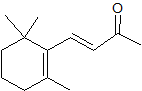 | [29] |
| ***Salsola komarovii* extract** | Mixture of several molecules with major bioactive components yet to be identified | no |  |  |  | + | Mixture of several molecules with major bioactive components yet to be identified | [30] |
| ***Rubra Radix***  **extract** | Mixture of several molecules with major bioactive components yet to be identified | Decreases GR binding to GREs |  |  |  |  | Mixture of several molecules with major bioactive components yet to be identified | [31] |
| **Synthetic analogues of natural compounds** | | | | | | | |  |
| **Compound A** | terpen | no | + | +/- |  | + | 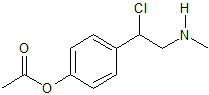 | [32] |
